# Lack of association of *TIM3 *polymorphisms and allergic phenotypes

**DOI:** 10.1186/1471-2350-10-62

**Published:** 2009-06-30

**Authors:** Jian Zhang, Denise Daley, Loubna Akhabir, Dorota Stefanowicz, Moira Chan-Yeung, Allan B Becker, Catherine Laprise, Peter D Paré, Andrew J Sandford

**Affiliations:** 1 James Hogg iCAPTURE Center for Cardiovascular and Pulmonary Research, St. Paul's Hospital, Vancouver, BC, Canada; 2 Respiratory Division, Department of Medicine, the University of Hong Kong, Hong Kong SAR; 3 The Section of Allergy and Clinical Immunology, Department of Pediatrics, University of Manitoba, Winnipeg, Canada; 4 Université du Québec à Chicoutimi, Saguenay, QC, Canada

## Abstract

**Background:**

T-cell immunoglobulin mucin-3 (TIM3) is a T_H_1-specific type 1 membrane protein that regulates T_H_1 proliferation and the development of immunological tolerance. TIM3 and its genetic variants have been suggested to play a role in regulating allergic diseases. Polymorphisms in the *TIM3 *promoter region have been reported to be associated with allergic phenotypes in several populations. The aims of this study were to examine whether genetic variation in the promoter region of *TIM3 *influenced transcription of the gene and risk for allergic phenotypes.

**Methods:**

We performed 5' rapid amplification of cDNA ends and reverse transcription-polymerase chain reaction. We screened for polymorphisms in the promoter region. Deletion analysis was used to localize the promoter region of *TIM3*. Genotyping was performed by TaqMan assays in three asthma/allergy population samples.

**Results:**

We found two regions with promoter activity in *TIM3*. One region was from -214 bp to +58 bp and the other from -1.6 kb to -914 bp relative to the transcription start site. None of the single nucleotide polymorphisms (SNPs) or haplotypes affected the transcriptional activity in reporter gene assays. No association between the SNPs and any phenotype was observed in the study cohorts.

**Conclusion:**

Our findings indicate that SNPs and haplotypes in the *TIM3 *promoter region do not have a functional effect *in vitro *and are not associated with allergic diseases. These data suggest that polymorphisms in the *TIM3 *promoter region are unlikely to play an important role in susceptibility to allergic diseases.

## Background

Asthma is a chronic inflammatory disease of the airways that is a major cause of morbidity in developed countries and has been increasing in prevalence [1,2]. Asthma is a common disease caused by interactions between multiple genes of small to modest effect and equally important environmental factors. Asthma susceptibility has been linked to several loci e.g. chromosomes 5, 6, 11, 12 and 14 [3]. Among these linkages, chromosome 5q23-35 has been replicated in several genome-wide studies in different populations [3].

McIntire *et al. *identified a chromosomal region that regulated T_H_2 cytokine production as well as airway hyperresponsiveness (AHR) using a congenic mouse model of asthma [4]. This region was distinct from the IL4 cytokine gene cluster and other nearby cytokine genes [4]. The region is homologous to human chromosome 5q33 and contains the *TIM *(T cell immunoglobulin domain and mucin domain) gene family [4]. There are two genes in this family (*TIM1 *and *TIM3*) that are biologically plausible atopy susceptibility genes. *TIM1 *(also known as the hepatitis A virus cellular receptor, *HAVCR1*) is expressed preferentially on T_H_2 cells and *TIM3 *(*HAVCR2*) is expressed preferentially on T_H_1 cells after activation of naive CD4^+ ^T-helper cells. T_H_1 cells mediate immune responses to intracellular pathogens, delayed-type hypersensitivity reactions, and produce cytokines such as interferon-γ, IL2, tumour-necrosis factor-α and lymphotoxin. T_H_2 cells mediate immune responses to extracellular pathogens and produce cytokines such as IL4, IL10 and IL13 which promote atopic and allergic diseases [5]. TIM1 promotes T_H_2 cytokine production and proliferation. In a murine model of asthma, stimulation of TIM1 in the presence of antigen prevented the development of respiratory tolerance and increased pulmonary inflammation [6]. TIM3 inhibits T_H_1-mediated auto- and alloimmune responses and acts via its ligand, galectin-9, to induce cell death in T_H_1 but not T_H_2 cells [7-9]. Considering their immunological function and chromosomal location both *TIM1 *and *TIM3 *are good candidate genes for asthma.

Recent association studies suggested that polymorphisms in the *TIM3 *promoter region may be associated with asthma-related phenotypes in both Caucasian and Asian population samples [10-12]. Other studies have demonstrated associations of *TIM1 *polymorphisms with asthma and related traits [11,13,14]. In the present study, we performed an association study in three asthma/allergy population samples to investigate the role of polymorphisms in the *TIM3 *promoter region and determined whether these polymorphisms affected *TIM3 *transcriptional activity.

## Methods

### Study populations

We used three independent asthma/allergy population samples: the Canadian Asthma Primary Prevention Study (CAPPS) cohort, the Study of Asthma Genes and the Environment (SAGE) birth cohort and the Saguenay-Lac-St-Jean (SLSJ)/Québec City (QC) Familial Collection (Table 1). The study protocols were approved by ethical review boards at all participating institutions. Informed consent was obtained from each individual or his/her guardian.

**Table 1:** Sample sizes by study, phenotype and ethnic background

	**Cohorts**			
	CAPPS	SAGE	SLSJ/QC	Combined
	
Families	545	723	306	1573
Genotyped	1316	1466	1234	4016

**Caucasian Samples (complete trios)**				

Phenotype				

Asthma	51	109	379	539
Atopy	105	145	362	612
AHR	142	96	278	516
Atopic Asthma	37	71	305	413

**Non Caucasian Samples^a^(complete trios)**				

Asthma	3	28	na	31
Atopy	18	44	na	62
AHR	14	22	na	36
Atopic Asthma	3	20	na	23

**Combined Analysis^b ^(complete trios)**				

Asthma	57	139	379	575
Atopy	135	190	362	687
AHR	170	120	278	568
Atopic Asthma	43	92	305	440

^a^Non Caucasian samples included individuals of Asian (Chinese, Korean and Japanese) and Canadian First Nations descent.

^b^Combined Analysis also contains trios with mixed and unknown ethnicity

CAPPS – Canadian Asthma Primary Prevention Study Cohort; SAGE – Study of Asthma Genes and the Environment birth cohort; SLSJ/QC – Saguenay – Lac-St-Jean/Québec City Familial Collection; AHR – airway hyperresponsiveness

The CAPPS cohort was initiated in 1995 and recruited from two Canadian cites, Vancouver and Winnipeg [15,16]. Infants were recruited who were at high risk for the development of asthma, defined as those who had at least one first-degree relative with asthma or two first-degree relatives with other allergic diseases. In total, there were 545 families recruited into this study (549 infants, 4 sets of twins). At the 7-year time point loss to follow-up was minimal, with 86% of the families completing a questionnaire. Spirometry and methacholine challenge testing were performed at the 7-year time point. The diagnoses of asthma and other atopic disorders were made by a pediatric allergist based on a detailed history and physical examination. Atopy was defined as at least one positive skin prick test. Methacholine challenge testing was carried out according to Cockcroft et al. [17]. The provocative concentration of methacholine that induced a 20% decrease in FEV_1 _from post-saline value (PC_20_) was determined. AHR for this cohort and the SAGE cohort was defined as a PC_20 _of less than 3.2 mg/ml methacholine [18,19].

SAGE is a population-based sample of 16,320 children, born in the province of Manitoba, Canada in 1995 [20]. In 2002, the families were sent a questionnaire to determine their health and home environment exposure. Children were classified according to the presence of asthma (n = 392), hay fever/food allergy (n = 192) or neither (n = 3002). All the children in the asthma and allergy groups were invited to participate in the study, together with a random sample (n = 200) of children with neither condition. A pediatric allergist assessed the presence of asthma based on a detailed history and physical examination, a methacholine challenge test was administered and skin prick tests for common allergens were performed. In total, 725 families were recruited into the study, including 247 with an asthmatic child and 328 with an atopic child.

The SLSJ/QC Familial Collection is comprised of 306 families from the Saguenay-Lac-Saint-Jean (n = 227) and Québec City (n = 79) regions of Québec, Canada [21,22]. There is at least one adult asthmatic proband in each family. Asthma was assessed using a respiratory health questionnaire and pulmonary function tests. AHR was defined as a PC_20 _< 8 mg/ml at the time of recruitment. If PC_20 _was not measurable, a 15% increase in FEV_1 _after inhalation of a bronchodilator or a variation in PEF of at least 12% within a 2-week period was also considered diagnostic of AHR. Participants were defined as having asthma if they had a reported history of asthma that was validated by a physician, or they showed asthma-related symptoms and a positive PC_20 _at the time of recruitment. Subjects were defined as atopic if they had at least one positive response to a skin prick test. Subjects with a PC_20 _> 8 mg/ml were considered not to have AHR; non-asthmatics were those who had no history of physician-diagnosed asthma, no symptoms of asthma and a PC_20 _greater than 8 mg/ml; non-atopics were those who had no positive response on skin prick test.

### Expression of *TIM3 *in tissues

The Human Multiple Tissue, Human Immune System cDNA Panels and Human Blood Fraction Panel (BD Biosciences/Clontech, Palo Alto, CA, USA) were used to analyze expression of *TIM3 *in various tissues. The PCR primers for the gene expression study are listed in Table 2. Resting CD14+ (monocytes), CD4+ (T helper/inducer cells), CD8+ (T suppressor/cytotoxic cells) and CD19+ (B lymphocytes) cells were positively selected from mononuclear cells from healthy donors by immunomagnetic separation with Dynabeads M-450 (Dynal, Oslo, Norway). Cells were activated with pokeweed mitogen (Invitrogen, San Diego, CA, USA) and concanavalin A (ICN, Costa Mesa, CA, USA) by standard methods, and the degree of activation of lymphocytes was estimated on the basis of morphological criteria (blast morphology and mitoses) and expression of two activation markers, CD25 (interleukin-2 receptor) and CD71 (transferrin receptor). We used glycerol-3-phosphate dehydrogenase (*G3PDH*) as an internal control for PCR. Amplification conditions were an initial denaturation step at 94°C for 10 min followed by 34, and 30 cycles of denaturation at 94°C for 30 s, annealing at 60°C for 30 s and extension at 72°C for 30 s for primer pairs amplifying *TIM3 *and *G3PDH*, respectively.

**Table 2:** Sequence of primers used in reverse transcriptase-polymerase chain reaction, 5' Rapid Amplification of cDNA ends (5'RACE) and (RT-PCR) and plasmid constructs

*TIM3 *RT-PCR primers	Forward	5'-tgctgctgctgctactacttaca-3'
	
	Reverse	5'-aggttggccaaagagatgag-3'
5'RACE	First round forward	5'-gctggggtgtagaagcagggcagat-3'
	
	First round reverse	5'-ccatcctaatacgactcactatagggc-3'
	
	Nested PCR forward	5'-tgtctgtgtctctgctgggccatgt-3'
	
	Nested PCR reverse	5'-actcactatagggctcgagcggc-3'

Plasmid constructs primers	Common reverse primer	5'-attatctcgagtggactgggtacttcttccaa
	
	Forward primer +63 bp	5'-attatggtacctgactgtagacctggcagtgtt-3'
	
	Forward primer -241 bp	5'-attatggtaccggacatgctccatttcaggt-3'
	
	Forward primer -452 bp	5'-attatggtacctgaggcttatgctgggagtt-3'
	
	Forward primer -914 bp	5'-attatggtaccaaaccactcagcctgtgagc-3'
	
	Forward primer -1702 bp	5'-attatggtaccgccttgaccaagttcatgct-3'
	
	Forward primer -2220 bp	5'-attatggtaccccagctccctacacacacaa-3'

### 5' Rapid Amplification of cDNA ends (5'RACE)

We performed 5' RACE experiments using commercially available RACE-ready human leukocyte and spleen cDNAs (Marathon Ready cDNA, BD Biosciences/Clontech) according to the manufacturer's instructions. Primers used for amplification for the first round PCR and for the nested PCR are shown in Table 2. The amplified RACE product was cloned into pCR2.1 TOPO-TA cloning vector (Invitrogen). Plasmids were purified by column chromatography (Invisorb Spin Plasmid Mini Kit, Invitek GmbH, Berlin) and subjected to direct sequencing with M13 primers.

### Single nucleotide polymorphism (SNP) screening and genotyping

Approximately 2500 bp of the 5' flanking region upstream of the transcription initiation site of *TIM3 *was amplified by PCR from genomic DNA of 19 unrelated healthy Caucasians. Subsequently, the products were subjected to direct sequencing with a Big-Dye Terminator Kit (Applied Biosystems, Foster City, CA, USA). Genotyping of the two tag SNPs was done by TaqMan Assay-on-Demand™ SNP typing (Applied Biosystems).

### Plasmid construction, transfection and luciferase assay

Genomic fragments of the 5' flanking region of exon 1 of *TIM3 *were amplified. PCR products were digested with *Xho*I and *Kpn*I overnight at 37°C and then subcloned into the pGL3-Basic vector (Promega, Madison, WI, USA) digested with *Xho*I and *Kpn*I. The clones were sequenced to confirm that the inserts were correct. The YT human T/NK cell line provided by Dr. Zacharie Brahmi as a gift was resuspended in RPMI 1640 (Sigma-Aldrich Co, St. Louis, MO, USA) with 20% FBS. Approximately 1 × 10^7 ^YT cells were cotransfected with 30 μg of test construct and 150 ng of pPL-TK (Promega) by electroporation with a Gene Pulsar II (Bio-Rad, Hercules, CA) set at 300 V and 975 μF. Transfected cells were harvested 24 h after transfection. Cells were lysed by the addition of 200 μl of lysis buffer (Promega). Twenty μl of each lysate was used for luciferase assay with the Dual-Luciferase Reporter Assay System (Promega). The firefly luciferase values were normalized to the *Renilla *luciferase values of pRL-TK, which were determined at the same time. The signal was read using a POLARstar OPTIMA (BMG, Alexandria, VA, USA) fluorimeter. Reporter activity is presented as the mean of at least five independent measurements.

### Statistical analysis

Differences in transcriptional activity in the reporter gene assays were analyzed by ANOVA and unpaired t-tests. We tested for association with asthma, atopy, atopic asthma and airway hyper- responsiveness phenotypes using the Family based Association Test (FBAT) software [23].

## Results

### Tissue expression of *TIM3*

**Figure 1:**
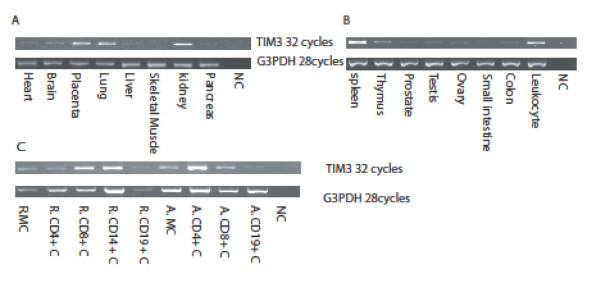
**Expression of *TIM3 *in multiple human tissues**. Results of PCR amplification of cDNA from different organs (A), the immune system (B), and blood fractions (C) are shown. *G3PDH *was included as an internal control. MC, mononuclear cells; R, resting; A, activated; NC, non-template control.

Expression of *TIM3 *was analyzed by PCR-based methods (Figure 1). *TIM3 *was strongly expressed in placenta, lung, kidney, spleen, and leukocytes. In the Human Blood Fraction Panel *TIM3 *was more highly expressed in active CD4+ cells than resting CD4+ cells. However it was more highly expressed in resting CD8+ cells than in active CD8+ cells. *TIM3 *was also strongly expressed in resting CD14+ cells. No splicing variants were found.

### Isolation of 5' full-length *TIM3 *transcripts and structure of the human *TIM3 *gene

**Figure 2:**
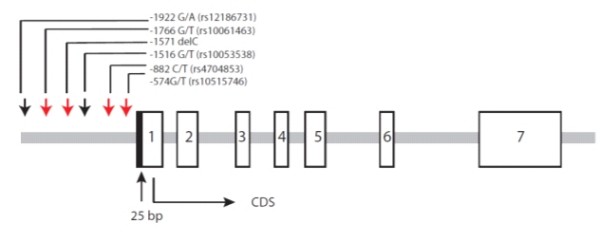
**Genomic structure of the human *TIM3 *gene**. The open boxes represent the positions of exons 1–7. The shaded box is the region we extended in our 5'RACE experiment. *TIM3 *contains seven exons and the coding sequence (CDS) starts in exon 1. The downward arrows indicate the SNPs in the promoter region. The four red arrows indicate SNPs that are in perfect LD.

Current information at the time of the experiment (January 2007) in the NCBI database  indicated that *TIM3 *was composed of seven exons and the translational start site was contained within exon 1. *TIM3 *was highly expressed in leukocytes and spleen and therefore 5' RACE experiments were performed with cDNAs derived from these cell types. We were able to identify an additional 25 bp of sequence on the 5' side of the known cDNA sequence (Figure 2). No additional novel exons were detected.

### Polymorphism screen

We screened for polymorphisms in the *TIM3 *promoter region using DNA from 19 unrelated normal subjects and found six polymorphisms -574 G/T (rs10515746), -882 C/T (rs4704853), -1516 G/T (rs10053538), -1571delC, -1766G/T (rs10061463) and -1922 G/A (rs12186731) in *TIM3 *(Figure 2). Among the six polymorphisms four (-574 G/T, -882 C/T, -1571delC and -1766G/T) were in perfect linkage disequilibrium (r^2 ^= 1). There were only three haplotypes formed by the six polymorphisms.

### Transcriptional activity of the 5' flanking region of *TIM3*

**Figure 3:**
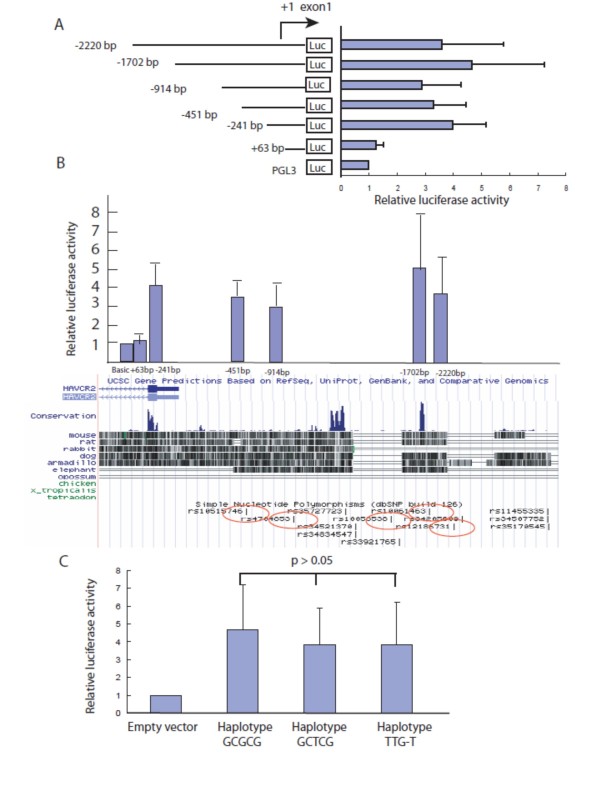
**Promoter activity assay of the human *TIM3 *gene promoter constructs**. (A) Luciferase activity is presented relative to the PGL3 basic vector after each construct was transfected into YT cells. All values are the mean ± SD of at least five independent experiments. (B) Comparison of the promoter activity and the conserved regions in the human genome. (C) Comparison of the promoter activity between the haplotypes.

To examine the transcriptional activity in the 5' flanking region of *TIM3*, we constructed plasmids that contained sequences from -2220, -1702, -914, -452, -241 and +63 relative to the transcription initiation site. The primers used for plasmid construction are listed in Table 2. The expression of *TIM3 *in YT cells was confirmed by RT-PCR (data not shown). The constructs were then transiently transfected into YT cells. Deletion analysis revealed that promoter activity of *TIM3 *in YT cells required at least 241 bp of upstream sequence and the maximal reporter gene expression was observed with the -1702 bp construct (Figure 3). There were five polymorphisms, -574 G/T, -882 C/T, -1516 G/T, -1571delC and -1766G/T in this region. To determine whether the five polymorphisms and their haplotypes were functional, we generated luciferase reporter gene constructs that contained the 5' flanking region of *TIM3 *from exon 1 to -1702 bp with three different haplotypes, i.e., haplotype GCGCG, haplotype GCTCG and haplotype TTG-T. The results showed there was no difference in expression level between each haplotype (Figure 3).

### FBAT analysis

To determine whether the previously reported *TIM3 *associations [10-12] were present in the CAPPS, SAGE and SLSJ/QC populations, we chose -882 C/T and -1922 G/A as tag SNPs. However -1922 G/A was in a region of repetitive sequence. Therefore, rs13170556, which was in perfect linkage disequilibrium (LD) with -1922 G/A, was genotyped in our samples. Both polymorphisms (rs13170556 and rs10061463) were in Hardy-Weinberg equilibrium (p > 0.1) in all cohorts. We performed FBAT analysis to test for association with asthma, atopy, atopic asthma and AHR. The results were corrected by the number of SNPs tested within *TIM3 *(n = 2) and the effective number of independent phenotypes (n = 3). We found that rs13170556 was associated with asthma in the CAPPS cohort in both the Caucasians only analysis and in the combined analysis of the Caucasian families with the non Caucasian families (p = 0.0138 and 0.0085, respectively) (Table 3). However, after correction for multiple testing we found no evidence for association in any of the three cohorts individually or in joint analysis of all the cohorts (Table 3). Similarly, there was no association found for -882C/T with any phenotype in any of the analyses (Table 4).

**Table 3:** Allele frequencies of the rs13170556 polymorphism in the three study cohorts for each phenotype

Cohort	Phenotype	Caucasian only				Combined analysis^a^			
		
		Frequency	OR	p	cp^c^	Frequency	OR	p	cp^c^
CAPPS	Asthma	0.13	0.31	0.0138	0.0822	0.13	0.29	0.0085	0.0508
					
	Atopy		0.73	0.3294	1.0000		0.62	0.1070	0.6359
					
	AHR		0.90	0.7455	1.0000		0.86	0.6393	1.0000
					
	Atopic Asthma		0.40	0.1031	0.6127		0.36	0.0653	0.3882

SAGE	Asthma	0.18	1.60	0.1477	0.8707	0.17	1.30	0.3757	1.0000
					
	Atopy		1.11	0.6961	1.0000		1.06	0.8084	1.0000
					
	AHR		1.33	0.3972	1.0000		1.15	0.6472	1.0000
					
	Atopic Asthma		1.80	0.1279	0.7540		1.43	0.3022	1.0000

SLSJ/QC	Asthma	0.15	0.95	0.7456	1.0000	0.15	0.95	0.7456	1.0000
					
	Atopy		0.86	0.3669	1.0000		0.86	0.3669	1.0000
					
	AHR		0.71	0.0749	0.4350		0.71	0.0749	0.4350
					
	Atopic Asthma		0.88	0.4758	1.0000		0.88	0.4758	1.0000

Combined analysis^b^	Asthma	0.15	0.94	0.6802	1.0000	0.15	0.91	0.5001	1.0000
					
	Atopy		0.89	0.3718	1.0000		0.86	0.2179	1.0000
					
	AHR		0.84	0.2402	1.0000		0.83	0.1775	1.0000
					
	Atopic Asthma		0.93	0.6434	1.0000		0.90	0.4961	1.0000

^a^Combined analysis of the Caucasian families with the non Caucasian families

^b^Combined analysis of the three cohorts (CAPPS, SAGE and SLSJ/QC)

^c^corrected p value

CAPPS – Canadian Asthma Primary Prevention Study Cohort; SAGE – Study of Asthma Genes and the Environment birth cohort; SLSJ/QC – Saguenay – Lac-St-Jean (SLSJ)/Québec City (QC) Familial Collection; AHR – airway hyperresponsiveness

**Table 4:** Allele frequencies of the rs10061463 polymorphism in the three study cohorts for each phenotype

Cohort	Phenotype	Caucasian only				Combined analysis^a^			
		
		Frequency	OR	p	cp^c^	Frequency	OR	p	cp^c^
CAPPS	Asthma	0.18	0.85	0.6829	1.0000	0.16	1.00	1.0000	1.0000
					
	Atopy		0.78	0.3859	1.0000		0.80	0.4137	1.0000
					
	AHR		0.87	0.5528	1.0000		0.86	0.4967	1.0000
					
	Atopic Asthma		0.55	0.2218	1.0000		0.73	0.4904	1.0000

SAGE	Asthma	0.17	1.10	0.7576	1.0000	0.15	1.13	0.6743	1.0000
					
	Atopy		1.18	0.5635	1.0000		1.14	0.6113	1.0000
					
	AHR		1.25	0.5045	1.0000		1.10	0.7576	1.0000
					
	Atopic Asthma		1.36	0.4319	1.0000		1.33	0.3972	1.0000

SLSJ/QC	Asthma	0.25	0.97	0.7902	1.0000	0.25	0.97	0.7902	1.0000
					
	Atopy		1.03	0.8263	1.0000		1.03	0.8263	1.0000
					
	AHR		1.12	0.4232	1.0000		1.12	0.4232	1.0000
					
	Atopic Asthma		1.00	1.0000	1.0000		1.00	1.0000	1.0000

Combined analysis^b^	Asthma	0.20	0.97	0.8149	1.0000	0.18	0.99	0.9542	1.0000
					
	Atopy		1.01	0.9526	1.0000		1.01	0.9542	1.0000
					
	AHR		1.07	0.5610	1.0000		1.05	0.6904	1.0000
					
	Atopic Asthma		0.99	0.9454	1.0000		1.02	0.8937	1.0000

^a^Combined analysis of the Caucasian families with the non Caucasian families

^b^Combined analysis of the three cohorts (CAPPS, SAGE and SLSJ/QC)

^c^corrected p value

CAPPS – Canadian Asthma Primary Prevention Study Cohort; SAGE – Study of Asthma Genes and the Environment birth cohort; SLSJ/QC – Saguenay – Lac-St-Jean (SLSJ)/Québec City (QC) Familial Collection; AHR – airway hyperresponsiveness

## Discussion

In the present study, we determined the expression pattern of *TIM3 *in human cells. We investigated the genomic structure and transcriptional activity of *TIM3 *and investigated polymorphisms in the promoter region of *TIM3 *in multiple cohorts. We isolated the full-length genomic region of *TIM3 *and characterized its promoter region. We found six polymorphisms in *TIM3*, but none was associated with asthma or the transcriptional activity of the gene *in vitro*.

*TIM3 *was initially cloned as a T_H_1-specific cell-surface marker. In our results, *TIM3 *was expressed on activated CD4+ cells as well as resting CD8+ cells and CD14+ cells, consistent with previous reports. In the mouse, *TIM3 *was expressed in both CD4+ and CD8+ cells [24,25] and in human peripheral blood mononuclear cells *TIM3 *was expressed at a higher level on CD14+ cells and CD8+ cells than on CD4+ cells [26]. *TIM3 *was also reported to be expressed in NK and NTK (NK-like T) cells [26,27]. In our results, *TIM3 *was expressed at a higher level in activated CD4+ cells than in resting CD4+ cells but conversely expression was higher in resting CD8+ than in activated CD8+ cells. Our results demonstrate that the expression level of *TIM3 *is not only differentially regulated in subsets of T cells but is also determined by the activation state of the cell.

*TIM3 *is expressed in human NK cells both at the mRNA and protein levels [26,27]. We found that *TIM3 *was also expressed in one type of NK cell line, the YT cell line, which was used in the reporter gene assays. We identified *TIM3 *promoter activity in the -241 bp and -1702 kb regions relative to the transcription initiation site. Conserved non-coding sequences may contain transcriptional regulatory elements participating in the temporal and tissue-specific expression patterns of genes [28,29]. In the UCSC website  there are three conserved regions in the *TIM3 *promoter (Figure 3B) and the first conserved region contributes to the -241 bp promoter region and the last two regions contribute to the -1702 bp promoter region. There are five SNPs in the -1.7 kb region and the -1516 G/T, -1571delC and -1766G/T SNPs flank the conserved sequence. However, the haplotype formed by these SNPs did not affect the promoter activity (Figure 3C). We also stimulated the YT cell line with IL-2 at different concentrations but we found no difference in promoter activity among the different haplotypes after the stimulation (data not shown).

There are discrepant reports concerning the association between *TIM3 *polymorphisms and allergic phenotypes [10-14]. Graves *et al*. [11] studied a mixed Caucasian/Hispanic population. The two *TIM3 *SNPs that showed association with eczema and atopy were rs1036199 and rs4704853. However, in our sample these two SNPs were in perfect LD and rs4704853 was not associated with any phenotype. This discrepancy may be due to the different ethnic group studied in the previous report [11]. Two other studies reported associations in Asian samples [10,12]. The -574G > T (rs10515746) polymorphism was associated with asthma and rhinitis in a Korean population although the -574T allele was found in less than 2% of the patients [10]. Therefore, our study may not have been adequately powered if this association is limited to the Asian population.

In Caucasian and African-American populations no association of *TIM3 *polymorphisms was seen with asthma or related phenotypes [13,14]. The three cohorts used in this study were family-based and there were more than 1000 individuals in each cohort. There were non-Caucasian samples in both SAGE and CAPPS but we analyzed the data separately to avoid possible loss of power due to genetic heterogeneity. Correction for multiple comparisons was performed to avoid false positive results. Although a nominal association of rs13170556 was found in the CAPPS cohort it was not significant after correction for multiple comparisons. Moreover, the association was not replicated in the other two cohorts and in the combined analysis of all three cohorts. Therefore, the association was likely a statistical artifact rather than a true positive result.

We did not analyze other phenotypes such as total or specific serum IgE in this study. We did not analyze haplotypes in the patient cohorts as we believe that this would have been inappropriate since we used tag SNPs from HapMap and it has been suggested that in this scenario there is little benefit of exhaustive haplotype testing [30]. In addition, we used the most powerful approach given our study design, there is high LD in the region, the marker coverage was not dense and our single SNP main effects were negative. All these factors made it unlikely that we would have benefited from haplotype tests.

The power to detect an association in this study varied with the phenotype, allele frequency and cohort considered. Power was calculated using the TDT Power Calculator [31]. For a major allele 'A' and minor allele 'a', we assumed the penetrance of the three genotypes was AA = 0.1, Aa = 0.2 and aa = 0.5. For an allele frequency of 0.13 and the phenotype of allergic asthma in the CAPPS cohort (i.e. 37 trios) the power to detect an association was only 0.41. However, for a sample size of 96 trios (e.g. AHR in the SAGE cohort) the power was 0.80 and was >0.80 for all other phenotypes in all cohorts in the Caucasians.

## Conclusion

Our findings indicate that SNPs and haplotypes in the *TIM3 *promoter region do not have a functional effect *in vitro *and are not associated with allergic diseases. These data suggest that polymorphisms in the *TIM3 *promoter region are unlikely to play an important role in susceptibility to allergic diseases.

## Competing interests

The authors declare that they have no competing interests.

## Authors' contributions

JZ participated in the genotyping, performed the remainder of the molecular analysis and produced the first draft of the manuscript, DD performed the analysis of the genetic epidemiological data and helped to draft the manuscript, LA participated in the genotyping, DS participated in the genotyping, MC-Y participated in the recruitment of the patient cohorts and helped to draft the manuscript, AB participated in the recruitment of the patient cohorts and helped to draft the manuscript, CL participated in the recruitment of the patient cohorts and helped to draft the manuscript, PDP participated in the recruitment of the patient cohorts, the design of the study and helped to draft the manuscript, AJS participated in the design of the study and helped to draft the manuscript. All authors read and approved the final manuscript.

## Pre-publication history

The pre-publication history for this paper can be accessed here:


